# The Relationship Between PSG and Morning/Evening Emotional Parameters in Patients With Insomnia Disorder and Good Sleepers

**DOI:** 10.3389/fpsyg.2018.02712

**Published:** 2019-01-10

**Authors:** Bernd Feige, Blanda Baumgartner, Dora Meyer, Dieter Riemann

**Affiliations:** Department of Clinical Psychology and Psychophysiology, Medical Center–University of Freiburg, Faculty of Medicine, University of Freiburg, Freiburg, Germany

**Keywords:** PSG (Polysomnography), insomnia, emotion, questionnaire, good sleepers

## Abstract

**Objectives and Introduction:** It is as yet unclear how polysomnographically determined sleep parameters determine emotional well-being both generally and particularly in patients with Insomnia Disorder (ID). ID is a frequent and disabling health condition associated with both day- and nighttime hyperarousal, linked to negative sleep-related ruminations as a cognitive component. Information on the immediate influence of objective sleep quality on emotional parameters is important for therapeutic approaches.

**Methods:** The relationship between objective sleep parameters and two emotional questionnaire items obtained both for evening and morning, relaxation and emotional balance, was determined for both sleep lab nights in 161 ID patients and 161 age and gender matched good sleepers (retrospective sample from the Freiburg data base, 98 female, 63 male in each group, age ID: 42.16 ± 11.55, GSC: 41.91 ± 11.30 years). Multivariate mixed effects analysis, corrected for global influences of group, age and first/second night, was employed to determine between- and within-subject influences of sleep and emotional parameters.

**Results:** Main effects: Within-subject, relaxation in the evening was strongly associated with sleep efficiency, REM latency and low arousal index in NREM sleep. No such influence was significant for emotional balance. Also between subjects, evening relaxation was related to increased sleep efficiency. Group interactions: Patients with larger relaxation values in the evening showed a larger reduction of the number of wake periods and the awakening index in NREM sleep than GSC subjects.

**Discussion:** Unexpectedly, no general influence of emotional balance on sleep was found. The subjective feeling of relaxation, however, was associated with sleep efficiency, REM latency and low NREM sleep arousal index. While the first association may be obvious, a direct link to REM latency and NREM arousal index has not previously been shown. We could also directly observe that the number of wake periods in the PSG is more strongly influenced by evening relaxation in ID patients than in good sleepers, asserting the importance of sleep perception and attitude toward sleep in the therapeutic process.

## Introduction

Theories of chronic insomnia emphasize the role of cognitive, emotional, and physiological hyperarousal for its development and maintenance (Harvey, 2002; Espie et al., 2006). Own work (Riemann et al., 2010, 2011, 2012, 2015) summarized that hyperarousal processes seem to play a key role in its pathophysiology. Autonomous, neuroendocrine, neuroimmunological, electrophysiological, neuroimaging, and psychological studies deliver converging evidence for increased levels of arousal in ID without comorbidity compared to good sleepers. Corresponding to the subjective experience of patients with insomnia having difficulties to “ shut down ” or to disengage from wakefulness (especially when trying to sleep), physiological data reflect increased levels of arousal both during day- and nighttime compared to good sleepers. It is assumed that the permanent hyperarousal in chronic insomnia—linked to habit formation or alternatively to an (epi-) genetic lack in the ability to down-regulate arousal (e.g., Palagini et al., 2014)—is triggered by stressful life events and maintained by sleep-preventing learned associations and maladaptive coping strategies (neurocognitive model of insomnia as formulated by Perlis et al., 1997).

Little is known on the concrete relationship between cognitive and emotional state in the evening and in the morning and objective measures of sleep. It is well-known that in Insomnia Disorder (ID) subjective sleep perception is often worse than objective sleep measures suggest, while healthy subjects tend to overestimate their sleep time (Carskadon et al., 1976; Frankel et al., 1976; Edinger and Fins, 1995; Means et al., 2003). This bad perception of sleep has been linked to qualitatively different REM sleep (Feige et al., 2008, 2018; Riemann et al., 2012). The studies on sleep perception are based upon subjective assessments in the morning after a sleep night. When assessing state variables such as cognitive and emotional state, however, two different aspects are equally important: The state at bedtime (evening) and the state change from evening to morning. The state at bedtime may influence sleep in the successive night, while the state change across the night may be influenced by the objective characteristics of sleep.

Sleep and affective phenomena have been linked before. People suffering from ID often do not feel refreshed and feel impaired in relevant areas of life as stated in DSM-IV (American Psychiatric Association, 2000) and DSM-5 (American Psychiatric Association, 2013). They also report worse mood than healthy controls and negative mood in ID correlates positively with subjective sleep latency (Buysse et al., 2007). Additionally, epidemiological studies show that insomnia increases the risk of developing depression (Baglioni et al., 2011) and anxiety disorders (Morphy et al., 2007; Neckelmann et al., 2007). A model by Walker (2009) allows to explain this relationship between sleep and affective disorders. He suggests that sleep plays a crucial role in emotion regulation and that memories and emotion are disentangled during sleep. If this disentanglement does not work properly, the risk of developing chronic anxiety and depression increases. Wassing et al. (2016) examined correlations between REM sleep disruption, hyperarousal, insomnia, and the resolution of emotional distress. They found a positive correlation between insomnia severity, restless REM sleep and duration of emotional distress (specifically shame) overnight. In the current study we aimed at the relationship between objective sleep parameters and more general emotional states as rated using the SF-A sleep questionnaire (Schlaffragebogen-A, Görtelmeyer, 1981).

This questionnaire is similar to a sleep diary in that it contains evening and morning items filled for each night. It measures subjective quality of sleep, feeling recuperated after sleep, psychological balance before going to sleep and psychosomatic symptoms during sleep. In addition, identical evening and morning items are available for relaxation and emotional balance. Combined with sleep laboratory examinations, it therefore allows to directly assess the relationship between the latter items—both state in the evening and change across the night with PSG (polysomnographic) parameters. Relaxation can be viewed as inverse arousal, thereby providing a link to the hyperarousal theories of insomnia. Emotional balance may provide an assessment of the importance of emotional processes.

Assessing two nights of every subject in addition allows us to discriminate between- and within-subject, i.e., trait- and state- like influences: Subjects generally reporting low emotional balance may, for example, show certain sleep characteristics by trait; this does not necessarily mean that targeting emotional balance therapeutically will change sleep as well. A within-subject relationship (between the nights) however, provides a better hint at a possible therapeutic pathway. At the same time we can control for a first night effect, which itself is thought to result from elevated arousal during the first night in a sleep laboratory (Agnew et al., 1966; Wauquier et al., 1991).

As most studies on sleep and emotion refer to subjective sleep data, the aim of this study was to explore potential relationships between subjective emotional states (relaxation and emotional balance) and objective sleep patterns in ID ad GSC using data from first and second night's sleep assessed at the sleep laboratory.

## Materials And Methods

### Participants and Procedure

#### Patients With Insomnia Disorder (ID)

This comparative observational study was based on a chart and data review of clinical patients with insomnia complaints evaluated between 1995 and 2012 at the sleep center of the Department of Psychiatry and Psychotherapy, Freiburg University Medical Center. During this period, 304 patients had been examined for two nights and diagnosed with Insomnia Disorder (ID; to ensure continuity, the diagnosis was primary insomnia after DSM-IV before DSM-5 and ID after DSM-5 thereafter, with exclusion criteria ensuring that this corresponded to primary insomnia after DSM-IV).

All patients had been referred from their primary care physician or medical specialist for evaluation of their sleep complaint. Two weeks before consultation in our outpatient sleep disorders clinic, patients received a questionnaire screening package by mail which included, among others, the Beck Depression Inventory (BDI, Beck and Steer, 1987; German version by Hautzinger et al., 1994) and the Pittsburgh Sleep Quality Index (PSQI, Buysse et al., 1989; German version by Riemann and Backhaus, 1996, see below). During the 1-h intake interview in the outpatient facility, patients were interviewed about: onset and duration of insomnia, sleep habits as well as history of medical illnesses, psychiatric disorders, use of medication, drugs, tobacco and alcohol, sleep disorders in first degree relatives (parents, siblings, and children) as well as education and social background.

A preliminary diagnosis was given on the basis of this interview and a decision was made about the necessity of a sleep laboratory examination (e.g., in case of: chronicity, persistence of insomnia despite adequate therapy, suspicion of possible underlying organic causes). Patients were then scheduled for a PSG evaluation approximately 4 to 8 weeks after the first outpatient contact.

During their 2-day stay in the sleep center, all patients underwent a thorough physical, psychiatric (repeating the interview taken 4–8 weeks earlier) and neurological examination, routine blood tests (blood cell count, liver, renal, and thyroid function), ECG, EEG, and urine drug screen (opiates, barbiturates, benzodiazepines, amphetamines and cannabis, and viral/bacterial infection).

Exclusion criteria for the present data analysis were: Presence of any other sleep disorder (e.g., sleep apnea syndrome, restless-legs syndrome, narcolepsy, circadian rhythm disorders, organic or psychiatric insomnia as defined by DSM-IV); A sleep apnea-/PLMS (period leg movements in sleep)-index with arousal ≥ 5.0/TST (total sleep time); Clinically relevant medical or neurological disorders or a positive urine drug screen; Consumption of hypnotic medication or medication known to affect sleep in the 2 weeks before or during sleep laboratory examination; Pregnancy; Any history of psychiatric disorder, of serious medical illness (e.g., hepatitis), substance abuse or shift work in the past.

During the two nights of sleep laboratory examinations patients had to refrain from alcohol. Decaffeinated coffee was only allowed in the morning for breakfast (maximum: two cups).

179 patients with confirmed insomnia disorder (ID) fulfilling the in- and exclusion criteria were finally eligible for statistical analysis.

#### Good Sleeper Controls (GSC)

One hundred and ninety-eight good sleeper controls (GSC) were available for the current study. They were selected retrospectively from our database of healthy subjects who participated in healthy volunteer studies of our sleep center. Control subjects underwent the same routine procedure of examinations as ID patients to ensure physical and psychiatric health. In addition to the exclusion criteria applying to the patients, good subjective sleep quality was required to be reflected in a PSQI sum score below 6. Medical problems including sleep apnea or restless legs syndrome were excluded. Written informed consent was obtained from all healthy subjects prior to the investigation in the sleep center.

#### Matched Groups for Final Analysis

From the ID and GSC groups described above, 161 ID patients and 161 GSC subjects could be matched using automated pair matching for gender and mean age within each gender group.

The final matched sample consisted of 63 men and 98 women of each group aged 42.16 ± 11.55 years (ID, 19–67 years) and 41.91 ± 11.30 years (GSC, 20–69 years). The age distribution did not differ (Wilcoxon W = 13122, *p* = 0.847).

### Polysomnography

All polysomnographic investigations were carried out using a standardized procedure. All subjects underwent two consecutive nights of PSG sleep monitoring. Sleep was recorded on 14-channel Nihon-Kohden EEG-polysomnographs for 8 h from “lights out” (23:00 h) until “lights on” (7:00 h) and digitized at a rate of 200 Hz. All recordings included EEG (C3A2; C4A1), EOG (horizontal and vertical) and EMG (submental) and were scored visually by experienced raters according to Rechtschaffen and Kales (1968) criteria. Inter-rater reliability is regularly evaluated and ensured to be above 0.9 as part of laboratory routine. During the first night, all subjects were screened for apneas and periodic leg movements by monitoring abdominal and thoracic effort, nasal airflow, oxymetry, and bilateral tibialis anterior EMG.

Sleep recordings were evaluated for the following parameters of sleep continuity and architecture: total sleep time (TST), sleep efficiency (SEI): ratio of TST to time in bed (TIB) x 100%; sleep onset latency (SOL): time from lights out until sleep onset (defined as first epoch of stage N2). Arousals were analyzed according to the criteria of the American Sleep Disorders Association (Sleep Disorders Atlas Task Force of the American Sleep Disorders Association, 1992). The arousal index is the number of arousals per hour. We evaluated both the arousal index within TST and sleep stage specific indices (stage N2, REM). In addition, short awakenings within N2 and REM sleep were captured accordingly as awakening index. Sleep architecture variables included: amounts of stages wake (W), N1, N2, slow wave sleep (SWS), and REM expressed as percentage of sleep period time (SPT: time from sleep onset until final awakening). REM sleep variables were REM latency (time from sleep onset until the first epoch of REM sleep, possible wake time not counted) and REM density, calculated as the ratio of 3 s REM mini-epochs including rapid eye movements (REMs) to the total amount of REM mini-epochs x 100%. REMs were defined using separate vertical and horizontal EOG traces, requiring a steepness of excursions of at least 70 μV/s.

### Subjective Sleep Scales

The PSQI (Buysse et al., 1989; Riemann et al., 1996) assesses sleep habits and quality in the preceding 2 weeks. Variables reported for group descriptives are the subjectively reported sleep onset latency, total sleep time, and (derived) sleep efficiency as well as the PSQI sum score (ranging from 0 to 21, highest values denoting severely impaired sleep).

The SF-A (Schlaffragebogen-A, Görtelmeyer, 1981, in its revised form Görtelmeyer, 2011) captures subjective aspects of sleep in the preceding night. It was administered in the morning after each sleep recording, after subjects were awake for some minutes. The questionnaire contains subjective estimates of wake times (SOL and wake after sleep onset, WASO) as well as the frequency of awakenings. Of the additional 5-level items (values 1-5) asked regarding to experiences in the evening prior to sleep and in the morning after sleep, “ relaxation ” and “ emotional balance ” are formulated identically for evening and morning and therefore can be directly compared. Both items are part of the SF-A factor scales “ psychological balancedness in the evening ” and “ feeling of recuperation in the morning. ” The German term “ Ausgeglichenheit ” translated here as “ balance ” or “ balancedness ” means absence of disturbing thoughts or emotions and could also be translated as “ calmness of the mind. ” The questions are “ how relaxed/how emotionally balanced did you/do you feel ”. The relationship of these two variables with PSG sleep is the major topic of this study. Descriptive subjective sleep quality data for both groups is given in Table 1.

**Table 1:** Descriptives of subjective sleep quality for both groups.

		**GSC**	**ID**	**Group**			**Age**		
		**Mean ± SD**	**Mean ± SD**	***B***	***F***	***p***	***B***	***F***	***p***
	Multivariate statistics (Wilk's Lambda)				0.36	**0.000**		0.85	**0.000**
PSQI	SOL	14.25 ± 10.93	41.89 ± 47.50	27.39	42.11	**0.000**	0.16	0.71	0.401
	TST	431.69 ± 56.28	302.17 ± 88.38	−126.30	216.97	**0.000**	−1.45	13.99	**0.000**
	SEI	87.79 ± 25.03	60.31 ± 26.19	−27.86	242.58	**0.000**	−0.16	4.20	**0.041**
	PSQI sum score	3.68 ± 2.12	10.96 ± 3.37	7.23	461.27	**0.000**	0.02	2.70	0.101
SF-A	SOL	11.35 ± 11.32	22.20 ± 17.90	10.86	36.50	**0.000**	0.08	1.00	0.317
	TST	452.92 ± 29.85	408.01 ± 61.61	−44.95	58.30	**0.000**	−0.29	1.20	0.274
	SEI	94.25 ± 6.13	84.90 ± 12.80	−9.35	58.43	**0.000**	−0.05	1.01	0.315
	SQ	3.58 ± 0.67	2.90 ± 0.74	−0.69	67.28	**0.000**	−0.01	3.03	0.083
	R_MOR	3.60 ± 0.72	2.80 ± 0.82	−0.84	82.36	**0.000**	0.01	5.35	**0.021**
	WB_EVE	3.92 ± 0.64	3.53 ± 0.66	−0.41	27.67	**0.000**	0.00	0.00	0.964
	EX_EVE	2.60 ± 0.67	2.92 ± 0.70	0.29	11.88	**0.001**	−0.01	2.99	0.085
	PS	1.49 ± 0.48	1.87 ± 0.47	0.38	52.92	**0.000**	0.01	13.50	**0.000**

*The SF-A of the second night is used. For the Group effect, B=ID-GSC. GSC, Good Sleeper Controls; ID Patients with Insomnia Disorder; PSQI, Pittsburgh Sleep Quality Index; min, Minutes; SOL, Sleep Onset Latency; TST, Total Sleep Time; SEI, Sleep Efficiency Index %; SF-A, Schlaffragebogen-A; SQ, Sleep Quality; R_MOR, Recovery in the morning; WB_EVE, Wellbeing in the evening; EX_EVE, Exhaustion in the evening; PS, Psychosomatic Symptoms. p values below p = 0.05 are set in bold font*.

### Statistical Analysis

Two-tailed non-parametric Wilcoxon tests were employed to ensure that the groups did not systematically differ in age. χ^2^ tests were used to compare dichotomous variables between groups. For descriptive purposes, means and standard deviations were calculated for PSG and subjective sleep parameters. Group differences (ID vs. GSC), night (first, second) and age effects were assessed using mixed-effects MANOVAs with between-subject factor GROUP and covariate AGE as well as within-subject factor NIGHT. Multivariate statistics were based on Wilk's Lambda. *P* < 0.05 was considered to be significant, proceeding from significant multivariate effects to univariate effects of the same independent variable. For univariate effects, we report *F* and *p* values as well as *B* values (betas) of the linear model, i.e., coefficients or differences between factor levels. For the NIGHT effect this is Night2-Night1 and for the GROUP effect ID-GSC.

For the main analysis of the influence of relaxation and emotional balance which were assessed before and after each night, mixed-effects MANOVAS were used with between-subject factor GROUP and covariate AGE as well as within-subject factor NIGHT and covariates morning values as well as changes across the night (morning-evening) of relaxation and emotional balance. Only the terms involving the target variables alone and their GROUP interactions are reported. The remaining terms are regarded as nuisance effects in this analysis. This pertains to the terms identical to the previous, more descriptive model (Table 2) and the NIGHT interactions.

**Table 2:** Characteristics of PSG data and the variables on relaxation and emotional balance.

	**Adaptation night**		**Baseline night**		**Effects**											
	**GSC**	**ID**	**GSC**	**ID**	**Group**			**Night**			**Night x Group**			**Age**		
	**Mean ± SD**	**Mean ± SD**	**Mean ± SD**	**Mean ± SD**	***B***	***F***	***p***	***B***	***F***	***p***	***B***	***F***	***p***	***B***	***F***	***p***
Multivariate statistics (Wilk's Lambda)						0.84	**0.000**		0.55	**0.000**		0.96	0.714		0.66	**0.000**
SOL (min)	22.87 ± 19.86	25.39 ± 22.10	17.44 ± 15.38	15.85 ± 11.97	0.45	0.08	0.778	−5.43	45.49	**0.000**	−4.11	3.42	0.065	0.05	0.55	0.461
TST (min)	391.71 ± 51.29	361.24 ± 64.06	419.22 ± 33.04	396.90 ± 47.24	−26.08	33.75	**0.000**	27.51	125.10	**0.000**	8.15	2.08	0.150	−1.29	41.57	**0.000**
SEI (%)	81.61 ± 10.58	75.35 ± 13.27	87.33 ± 6.85	82.67 ± 9.81	−5.40	33.71	**0.000**	5.72	125.86	**0.000**	1.61	1.92	0.167	−0.27	42.61	**0.000**
NWP	29.27 ± 13.80	31.68 ± 14.95	27.50 ± 14.34	29.56 ± 14.57	2.14	2.65	0.105	−1.77	8.41	**0.004**	−0.35	0.07	0.795	0.39	42.72	**0.000**
W (%SPT)	12.95 ± 9.08	17.92 ± 11.92	8.49 ± 5.61	12.68 ± 9.39	4.52	30.85	**0.000**	−4.46	83.18	**0.000**	−0.78	0.54	0.461	0.26	50.73	**0.000**
N1 (%SPT)	9.30 ± 4.92	9.98 ± 4.92	8.02 ± 4.65	8.64 ± 4.22	0.62	2.05	0.153	−1.28	44.32	**0.000**	−0.06	0.02	0.877	0.14	47.00	**0.000**
N2 (%SPT)	51.96 ± 8.43	50.39 ± 9.51	54.93 ± 7.24	53.04 ± 9.12	−1.71	4.04	**0.045**	2.98	44.52	**0.000**	−0.32	0.14	0.706	−0.07	3.38	0.067
N3 (%SPT)	7.02 ± 6.55	5.05 ± 5.81	7.97 ± 6.78	6.24 ± 7.06	−1.80	8.01	**0.005**	0.94	26.04	**0.000**	0.24	0.33	0.566	−0.20	50.39	**0.000**
REM (%SPT)	18.44 ± 5.44	16.55 ± 5.44	20.39 ± 4.91	19.25 ± 4.57	−1.49	10.10	**0.002**	1.94	71.57	**0.000**	0.76	1.90	0.169	−0.12	31.20	**0.000**
REML (min)	101.51 ± 48.39	107.84 ± 60.51	76.50 ± 33.55	72.94 ± 30.44	1.34	0.13	0.719	−25.01	86.81	**0.000**	−9.89	2.37	0.125	0.16	0.88	0.348
REMD (%)	24.96 ± 7.51	25.97 ± 8.77	25.36 ± 7.83	26.94 ± 7.41	1.30	2.61	0.107	0.40	3.54	0.061	0.58	0.63	0.429	−0.04	1.32	0.252
AI/REM	18.19 ± 9.32	18.50 ± 9.38	17.59 ± 9.10	17.76 ± 9.03	0.22	0.07	0.795	−0.60	2.41	0.121	−0.14	0.03	0.872	0.09	4.47	**0.035**
AI/N	12.30 ± 6.23	13.72 ± 7.95	10.33 ± 5.52	10.91 ± 6.07	0.98	2.28	0.132	−1.97	74.12	**0.000**	−0.85	2.34	0.127	0.09	9.83	**0.002**
AWI/REM	3.49 ± 3.78	3.66 ± 3.33	3.58 ± 3.69	3.75 ± 3.46	0.16	0.25	0.620	0.09	0.24	0.624	−0.01	0.00	0.985	0.06	13.94	**0.000**
AWI/N	3.90 ± 1.78	4.37 ± 2.20	3.38 ± 1.76	3.68 ± 1.89	0.38	4.67	**0.031**	−0.52	39.24	**0.000**	−0.16	0.73	0.393	0.05	39.06	**0.000**
Multivariate statistics (Wilk's Lambda)						0.76	**0.000**		0.91	**0.000**		0.96	**0.019**		0.96	**0.006**
Relaxation Evening	3.80 ± 0.77	3.22 ± 0.90	3.84 ± 0.81	3.48 ± 0.80	−0.47	36.28	**0.000**	0.04	9.19	**0.003**	0.21	4.61	**0.033**	0.00	0.34	0.560
Em.Balance Evening	3.87 ± 0.78	3.47 ± 0.87	3.93 ± 0.83	3.45 ± 0.91	−0.44	29.63	**0.000**	0.06	0.19	0.662	−0.07	0.47	0.492	0.01	3.54	0.061
Relaxation M-E	−0.09 ± 0.91	−0.49 ± 1.12	−0.01 ± 0.90	−0.39 ± 0.90	−0.39	22.23	**0.000**	0.08	1.71	0.192	0.02	0.02	0.892	0.00	1.65	0.200
Em.Balance M-E	−0.10 ± 0.85	−0.47 ± 1.04	0.01 ± 0.81	−0.16 ± 0.95	−0.27	11.06	**0.001**	0.11	10.53	**0.001**	0.20	2.55	0.111	0.01	3.16	0.077

*For the Group effect, B=ID-GSC. For the Night effect, B=Night 2 - Night 1. p values below p = 0.05 are set in bold font*.

The rationale for using evening scores and differences across the night instead of evening and morning scores was that evening and morning scores can be expected to be related to some degree; also, the difference across the night can be hypothesized to be determined by some property of the intervening sleep. If evening and morning scores were entered independently into an analysis, this important change aspect would be reflected only in an interaction term of these covariates, rendering analysis and conclusions more complex.

All statistical analyses were performed using the statistical software suite “ R ” version 3.5.1 (R Core Team, 2018).

## Results

### Main Effects for Group, Night, and Age

Table 2 shows the more descriptive MANOVAs separately for the PSG variables and the target variables relaxation and emotional balance. For PSG, multivariate effects are seen for GROUP, NIGHT, and AGE but not NIGHT x GROUP. The GROUP effect shows the typical reductions in total sleep time (TST) and sleep efficiency index (SEI) in the ID group as well as an increased awakening index in NREM sleep. The NIGHT effect shows reduced sleep onset latency (SOL), increased TST and SEI, reduced number of wake periods (NWP) as well as arousal and awakening indices in NREM sleep (AI/N and AWI/N) in the second relative to the first night. Increased AGE is associated with reduced TST and SEI, increased NWP as well as increases in arousal and awakening indices in both NREM and REM sleep.

### Target Variables (Relaxation and Emotional Balance)

For the target variables (lower part of Table 2), all multivariate effects are significant. Patients with ID show reductions in all four variables (relaxation and emotional balance, both evening values and differences morning-evening). In the second night, relaxation in the evening and the emotional balance difference morning-evening are increased. As evidenced by the NIGHT x GROUP effect, the increase in evening relaxation across nights was significantly higher in ID patients. Finally, age tended to increase both emotional balance in the evening and its morning-evening difference, not reaching significance.

#### Between-Subject Effects

**Figure 1:**
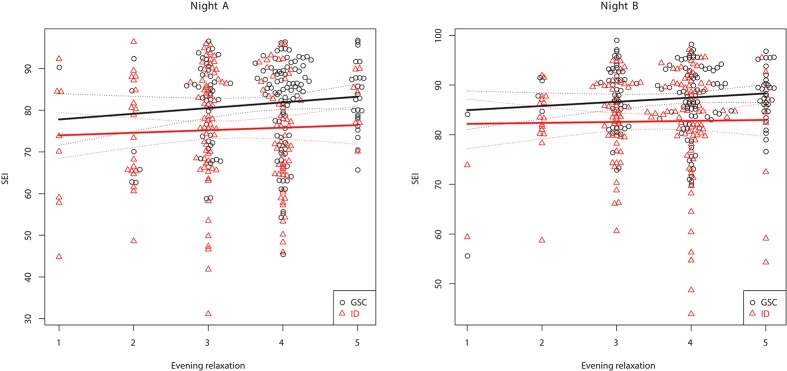
Relationship between evening relaxation and SEI (%) for the two groups in first **(Left)** and second **(Right)** nights.

**Figure 2:**
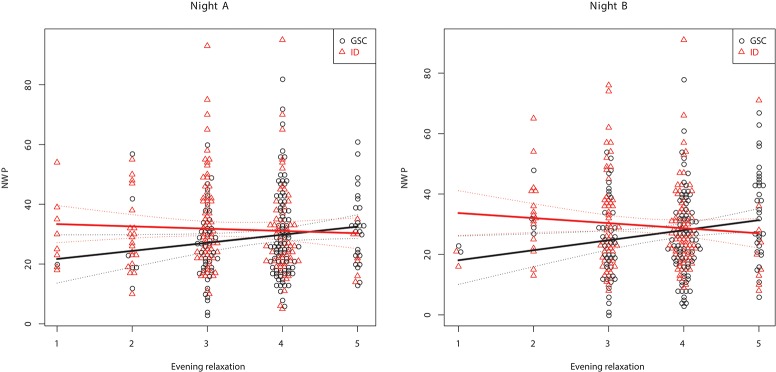
Relationship between evening relaxation and NWP for the two groups in first **(Left)** and second **(Right)** nights.

Table 3 shows the between-subject relationships between the target variables and PSG, i.e., whether subjects with different sleep characteristics tend to also show different values on relaxation and emotional balance. Multivariate significance is seen for evening relaxation and the evening relaxation x GROUP interaction. Subjects with larger evening relaxation show reduced SOL and increased TST, SEI, and stage N3 % SPT. Patients with larger evening relaxation show reduced NWP and awakening index in NREM sleep (AW/N) relative to the control group. Figures 1, 2 show the relationship between evening relaxation and SEI as well as NWP, respectively.

**Table 3:** Between-subject effects of relaxation and emotional balance variables.

	**Between**																							
	**Evening**						**Morning–Evening**						**Evening x Group**						**Morning–Evening x Group**					
	**Relaxation**			**Emotional balance**			**Relaxation**			**Emotional balance**			**Relaxation**			**Emotional balance**			**Relaxation**			**Emotional balance**		
	***B***	***F***	***p***	***B***	***F***	***p***	***B***	***F***	***p***	***B***	***F***	***p***	***B***	***F***	***p***	***B***	***F***	***p***	***B***	***F***	***p***	***B***	***F***	***p***
Multivariate statistics (Wilk's Lambda)		0.92	**0.049**		0.92	0.064		0.98	0.963		0.95	0.405		0.90	**0.014**		0.95	0.407		0.95	0.389		0.94	0.211
SOL (min)	−20.44	10.66	**0.001**	13.99	0.05	0.829	−11.51	0.14	0.708	−2.10	1.46	0.227	−13.53	1.72	0.191	12.73	0.19	0.665	−4.89	1.44	0.231	22.86	0.30	0.584
TST (min)	32.15	8.28	**0.004**	−20.01	1.68	0.195	−4.63	1.14	0.287	34.51	0.59	0.443	62.94	0.97	0.325	−57.56	3.63	0.058	67.81	0.28	0.597	−58.81	0.22	0.639
SEI (%)	6.13	7.87	**0.005**	−4.58	1.53	0.218	−1.05	1.17	0.281	6.91	0.48	0.490	13.94	0.76	0.383	−11.13	3.82	0.052	13.96	0.33	0.568	−11.54	0.28	0.597
NWP	−0.82	0.08	0.779	13.46	0.00	0.945	3.04	0.10	0.747	3.35	0.03	0.854	−11.95	5.59	**0.019**	−12.59	5.17	**0.024**	−12.42	1.93	0.165	−2.95	0.02	0.896
W (%SPT)	−0.64	2.90	0.089	−0.08	1.47	0.226	3.61	1.50	0.222	−8.71	0.20	0.653	−11.74	0.16	0.691	9.21	4.45	**0.036**	−12.63	0.07	0.793	9.63	1.95	0.164
N1 (%SPT)	−2.07	2.52	0.113	0.66	0.34	0.558	2.08	0.01	0.926	−2.05	0.02	0.879	1.17	1.96	0.162	−1.38	0.86	0.356	−2.99	1.57	0.212	0.55	1.84	0.176
N2 (%SPT)	−3.82	0.01	0.909	−2.56	0.26	0.613	−5.35	0.65	0.422	0.11	6.14	**0.014**	11.26	0.03	0.860	−5.49	6.95	**0.009**	14.06	0.03	0.868	−4.23	0.12	0.728
N3 (%SPT)	3.47	5.85	**0.016**	1.01	0.91	0.340	1.02	0.10	0.747	5.53	6.70	**0.010**	−2.04	1.58	0.210	3.15	0.23	0.633	−2.11	1.45	0.230	−2.98	0.01	0.921
REM (%SPT)	2.66	1.61	0.205	0.81	0.04	0.843	−1.30	0.84	0.360	4.26	0.02	0.892	1.42	0.61	0.434	−4.96	0.37	0.545	3.43	0.06	0.806	−1.94	0.28	0.597
REML (min)	8.77	0.71	0.401	−29.24	1.88	0.171	−9.95	0.53	0.468	−15.46	0.09	0.763	−40.13	2.23	0.137	39.68	0.16	0.687	−23.79	0.98	0.324	−6.26	1.23	0.268
REMD (%)	−10.69	0.51	0.475	3.31	2.89	0.090	−9.03	0.75	0.386	3.30	0.14	0.712	4.59	2.76	0.098	−9.80	0.40	0.530	2.11	1.31	0.252	−2.11	0.16	0.691
AI/REM	−3.80	1.19	0.276	5.89	0.33	0.566	−5.26	0.00	0.954	8.23	0.23	0.630	11.28	0.83	0.364	−3.03	0.49	0.483	8.18	1.58	0.210	−10.00	0.03	0.873
AI/N	−2.90	0.18	0.673	5.04	9.32	**0.002**	0.22	0.57	0.452	−0.39	0.07	0.797	−7.50	1.35	0.246	3.15	3.09	0.080	−4.57	2.53	0.113	−3.14	0.02	0.888
AWI/REM	0.83	0.45	0.504	2.87	2.40	0.122	0.40	0.04	0.845	0.97	0.02	0.887	−1.70	0.64	0.423	−2.86	2.35	0.126	−0.66	0.16	0.689	0.34	0.01	0.908
AWI/N	−0.86	0.12	0.730	1.87	0.87	0.353	0.25	0.50	0.481	−0.02	0.45	0.502	−1.72	4.31	0.039	−1.07	4.03	0.046	−2.11	1.26	0.262	0.26	0.00	0.988

*p values below p = 0.05 are set in bold font*.

#### Within-Subject Effects

Table 4 shows the within-subject relationships between the target variables and PSG, i.e., whether nights with different sleep characteristics within the same subject tend to show different values on relaxation and emotional balance. This is generally more valuable than between-subject effects, since possible different response tendencies across subjects are factored out and within-subject effects are more suitable to predict treatment effects.

**Table 4:** Within-subject effects of relaxation and emotional balance variables.

	**Within**																							
	**Evening**						**Morning–Evening**						**Evening x Group**						**Morning–Evening x Group**					
	**Relaxation**			**Emotional balance**			**Relaxation**			**Emotional balance**			**Relaxation**			**Emotional balance**			**Relaxation**			**Emotional balance**		
	***B***	***F***	***p***	***B***	***F***	***p***	***B***	***F***	***p***	***B***	***F***	***p***	***B***	***F***	***p***	***B***	***F***	***p***	***B***	***F***	***p***	***B***	***F***	***p***
Multivariate statistics (Wilk's Lambda)		0.84	**0.000**		0.96	0.608		0.78	**0.000**		0.94	0.216		0.94	0.209		0.95	0.476		0.95	0.369		0.95	0.541
SOL (min)	−7.48	15.25	**0.000**	1.69	2.38	0.124	1.53	6.91	**0.009**	−2.92	0.58	0.445	2.78	1.39	0.240	0.93	1.42	0.234	1.60	0.70	0.404	7.48	0.89	0.345
TST (min)	17.49	12.08	**0.001**	−1.14	2.56	0.111	−2.66	29.05	**0.000**	8.90	4.55	**0.034**	6.47	3.90	**0.049**	−28.64	1.21	0.272	7.87	2.90	0.090	−12.99	0.15	0.699
SEI (%)	3.24	12.06	**0.001**	0.05	2.50	0.115	−0.93	31.70	**0.000**	2.11	4.47	**0.035**	2.22	3.51	0.062	−6.45	1.76	0.186	2.00	3.35	0.068	−3.02	0.13	0.718
NWP	0.08	0.09	0.771	−0.86	0.02	0.895	3.96	8.72	**0.003**	−5.38	1.70	0.194	−4.80	0.76	0.384	5.77	0.44	0.509	−9.67	1.50	0.222	6.99	1.89	0.170
W (%SPT)	−0.68	3.04	0.082	−1.12	0.79	0.375	1.24	33.53	**0.000**	−2.48	0.96	0.329	−5.90	3.35	0.068	9.40	0.07	0.785	−4.16	4.42	**0.036**	3.78	0.85	0.358
N1 (%SPT)	−1.00	10.62	**0.001**	2.03	0.15	0.699	0.05	4.30	**0.039**	0.46	2.74	0.099	−0.29	1.39	0.240	−1.21	0.73	0.392	−1.03	1.72	0.190	0.62	3.31	0.070
N2 (%SPT)	2.56	3.07	0.081	−0.78	1.57	0.212	−0.32	26.12	**0.000**	0.28	0.04	0.844	2.89	2.75	0.098	−4.40	0.38	0.541	4.11	1.07	0.302	−2.25	0.10	0.751
N3 (%SPT)	−1.70	0.00	0.999	0.46	0.60	0.438	−0.37	5.72	**0.017**	1.84	0.94	0.334	2.64	1.07	0.302	−1.64	0.01	0.910	0.35	0.32	0.573	−1.58	0.07	0.798
REM (%SPT)	0.37	8.54	**0.004**	−0.15	0.60	0.439	−0.82	7.85	**0.005**	0.07	2.50	0.115	1.00	6.35	**0.012**	−2.58	1.37	0.244	0.90	8.55	**0.004**	−0.73	0.46	0.499
REML (min)	7.36	10.28	**0.001**	−5.38	0.03	0.869	18.87	16.62	**0.000**	−16.70	2.11	0.147	−49.62	2.69	0.102	30.47	0.82	0.365	−12.41	4.59	**0.033**	8.09	0.02	0.885
REMD (%)	−2.33	3.86	0.050	−0.19	1.34	0.248	−0.55	0.15	0.696	2.65	0.43	0.510	2.43	0.71	0.400	2.74	1.04	0.309	1.39	4.16	**0.042**	−3.00	0.73	0.394
AI/REM	4.25	1.23	0.268	−1.85	0.00	0.975	−0.06	0.03	0.858	−0.30	0.36	0.551	−7.13	0.11	0.738	4.27	0.00	0.979	−2.65	0.37	0.541	3.48	0.54	0.465
AI/N	−1.10	20.80	**0.000**	0.46	2.37	0.125	0.19	14.21	**0.000**	−1.41	1.46	0.228	−3.28	1.21	0.272	3.04	0.11	0.737	−0.74	5.22	**0.023**	0.84	0.00	0.997
AWI/REM	2.20	3.94	**0.048**	−1.65	0.71	0.400	1.53	2.82	0.094	−0.80	0.21	0.648	−1.89	0.20	0.657	2.05	0.00	0.994	−1.55	1.34	0.248	1.15	0.17	0.682
AWI/N	−0.36	3.69	0.056	0.07	0.21	0.651	0.43	17.06	**0.000**	−0.58	1.50	0.221	−0.53	4.06	**0.045**	0.86	0.07	0.790	−1.30	0.76	0.385	1.01	3.03	0.083

*p values below p = 0.05 are set in bold font*.

Across groups, relaxation in the evening was strongly related to within-subject PSG sleep quality: Reduced SOL, increased TST and SEI as well as REM % SPT and REM latency (REML) and a reduced arousal index in NREM sleep were related to increased evening relaxation. The awakening index in REM sleep was, surprisingly, slightly positively related to evening relaxation.

**Figure 3:**
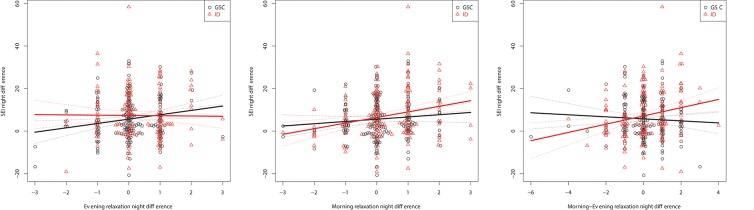
Within-subject relationships to the SEI captured as differences between nights: Evening **(Left)**, Morning **(Middle)**, and Morning-Evening **(Right)** relaxation.

Unexpectedly, the influence of the Morning-Evening relaxation difference is for the most part of opposite direction to the evening value, albeit with lower amplitude. This indicates that evening relaxation is more important than morning relaxation; with the scores limited between 1 and 5, a positive Morning-Evening difference can only be attained if the evening relaxation value is less than 5, associated with a negative impact on sleep quality. Figure 3 shows these relationships graphically for the example of SEI.

No other main effects or interactions reached significance, particularly no relationship could be found between the scales of emotional balance and any PSG sleep parameter.

## Discussion

In the current study, we reported an extensive multivariate analysis of the relationship between relaxation and emotional balance in a large population of patients with insomnia and matched healthy controls.

The level of relaxation, particularly in the evening, was found to enhance sleep both between subjects and within subjects. This suggests that relaxation can be an important therapeutic target for treating sleep problems.

A single significant group interaction was identified: Evening relaxation reduced the number of wake periods and, specifically, the awakening index in NREM sleep more strongly in patients with insomnia disorder. In fact, relaxation techniques are important components in current cognitive-behavioral treatments for insomnia (CBT-I, Trauer et al., 2015; Riemann et al., 2017a,b; Friedrich and Schlarb, 2018), although clearly only responsible for part of its therapeutic efficacy (Norell-Clarke et al., 2015). Bertisch et al. (2012) have found that relaxation techniques are generally under-used for sleep problems in the general population. However, the therapeutic effect of relaxation alone may not be sustainable, requiring other components of CBT-I for a lasting effect.

The level of emotional balance as assessed by the SF-A could not be linked to objective sleep parameters in this multivariate analysis. This is interesting by and of itself, as ID patients showed clearly reduced levels of emotional balance as a group, an effect which has been partialled out of the emotional balance—PSG relationships in our analysis. Therefore, the finding means that, within each group, differences in emotional balance were related to PSG parameters neither between- nor within subjects. It is well-known that emotional reactivity is impaired in insomnia, as well as some aspects of emotional valence (sleep in good sleepers being distinguished by increased positive but not necessarily reduced negative emotions; Baglioni et al., 2010). Since these were group studies comparing insomnia patients to good sleepers and the current study found clear group differences in SF-A emotional balance as well, it may still be that the latter construct captures the deficiencies in emotional processing characteristic to insomnia to some degree. In this case the current finding could be extended to emotional reactivity and valence as well. This in turn would indicate that targeting emotional reactivity, valence or balance itself would rather not present a viable therapeutic approach. Further studies making this link explicit are, however, needed to support this conclusion.

Generally, mutual relationships between sleep and emotions have to be considered (Kahn et al., 2013). Complicating the matter further, reappraisal processes may be involved (cf. Palmer and Alfano, 2017), for example general dissatisfaction with sleep leading to more negative responses to any sleep-related question. This could be a potential mechanism for the more negative judgments on both the relaxation and emotional balance scale in ID patients (Table 3).

Wassing et al. (2016) reported that the overnight resolution of emotional distress contributes to hyperarousal. They specifically targeted shame. Overnight resolution of emotional distress means an amelioration of values during sleep (positive Morning-Evening difference in our study). We did not see an influence of emotional balance (or its difference across the night) on sleep parameters, but a measure of (inverse) hyperarousal (i.e., relaxation) was included in the same model and apparently correlated better with objective sleep parameters than emotional balance itself. Thus, for the SF-A emotional balance construct it appears that its change across the night is rather not related to hyperarousal or objective sleep parameters. Since there is a clear group difference in emotional balance, it is possible that impaired emotional balance over longer periods of time (e.g., weeks) leads to increased hyperarousal. This notion cannot be tested using the data of the current study but should be addressed by future studies.

In summary, in the current study we could separately assess state- and trait- like influences of two emotional parameters, relaxation and emotional balance, on PSG in large matched samples of patients with ID and good sleeper controls. While both parameters were lower in ID patients (“ trait ”), particularly increased evening relaxation had a strong within-subject influence on PSG sleep quality as a main effect across both groups and was additionally linked to a reduced number of wake periods in the ID patients, suggesting relaxation as a useful therapeutic target in conjunction with other CBT-I elements.

## Limitations

While interpreting the results of this study some limitations should be taken into account. First of all we used pre-existing data which on the one hand lead to a large sample size but on the other hand restricted the measurement of the emotional state to two subjective items without defined valence. To assess the emotional state more broadly, another procedure should be chosen in future studies. Buysse et al. (2007) presented a possible procedure that additionally avoids ground effects. Furthermore the evening data as used here was assessed in the following morning and could therefore be influenced by the morning emotional state. To avoid such bias future studies should assess the emotional state in the actual evening. Possibly, more questionnaires for sample description would have been useful.

Just like in every other sleep laboratory study the ecological validity of the results can be questioned. The time needed for adjustment to the changed sleep environment is not known exactly, although an adaption phase of one night is usually assumed (Le Bon et al., 2001). Since good signal quality and monitoring options are still lacking for the home environment, sleep laboratory data in general may lack ecological validity. This is, however, partially true also for sleep studies conducted at home, because the presence of the recording equipment is a factor in the “ First Night Effect ” as well (Blackwell et al., 2017). In the current study we analyzed both between- and within-subject influences between PSG and emotional parameters. The “ First Night Effect ” generates welcome within-subject variability in this respect (it can be interpreted as a “ stress probe ” for sleep). Part of our within-subject results could be due to this special situation with an unusually disturbed first night.

Finally, most of our data has been collected during clinical routine. Therefore characteristics like personality traits or chronotype have not been assessed systematically for all subjects although these characteristics could be related to potential subgroup-differences and should therefore be assessed in future studies.

## Ethics Statement

The current study is a retrospective analysis of studies carried out in accordance with the recommendations of the ethics committee of the University of Freiburg Medical Center with written informed consent from all subjects. All subjects gave written informed consent in accordance with the Declaration of Helsinki. The protocol was approved by the ethics committee of the University of Freiburg Medical Center.

## Author Contributions

BF and DR devised the study. BF devised the data analysis, interpreted the data and wrote the manuscript. BB and DM analyzed and interpreted the data. All authors contributed to the manuscript.

### Conflict of Interest Statement

The authors declare that the research was conducted in the absence of any commercial or financial relationships that could be construed as a potential conflict of interest.
